# Mechanisms and traditional Chinese medicine therapeutics for primary osteoporosis: an integrated perspective

**DOI:** 10.3389/fendo.2025.1638629

**Published:** 2025-08-08

**Authors:** Zhongcheng An, Bing Wu, Wangnan Mao, Lianguo Wu

**Affiliations:** ^1^ Department of Orthopedics, Second Affiliated Hospital of Zhejiang Chinese Medical University, Hangzhou, Zhejiang, China; ^2^ The Second Clinical College, Zhejiang Chinese Medical University, Hangzhou, China

**Keywords:** primary osteoporosis, etiology and pathogenesis, pathogenesis, Chinese medicine therapeutics, treat

## Abstract

Primary osteoporosis (POP) is a systemic skeletal disorder characterized by compromised bone microarchitecture. With the progression of population aging trends, POP has garnered increasing societal concern as a major public health challenge. Contemporary research reveals that POP pathogenesis involves intricate interactions among endocrine regulation, genetic predisposition, and neuromodulation pathways. Recent advances in Traditional Chinese Medicine (TCM) investigations have demonstrated its unique therapeutic advantages in POP management through multi-target regulatory mechanisms. This review systematically examines the molecular pathogenesis of POP and deciphers potential therapeutic mechanisms of TCM for POP involving bone metabolism regulation, including herbal compound-induced osteoblast activation, osteoclast inhibition, and extracellular matrix remodeling. By integrating evidence from pharmacokinetic studies and clinical trials, this analysis provides scientific validation for the efficacy and pharmacological rationale of TCM interventions, while proposing novel clinical intervention strategies that synergize traditional therapeutic wisdom with modern precision medicine approaches. This comprehensive review was conducted based on a systematic literature search across PubMed, Web of Science, China National Knowledge Infrastructure (CNKI), and Wanfang Data databases, encompassing original research articles and reviews published between 2004 and 2024. The search focused on the pathogenesis of primary POP (including hormonal regulation, genetic factors, neuromodulation) and the therapeutic effects of Traditional Chinese Medicine (TCM) (single herbs and formulas). Studies were selected according to predefined inclusion (clear relevance, robust methodology) and exclusion criteria (case reports, duplicates, low-quality studies). Key findings from the included literature were synthesized and critically analyzed.

## 1 Introduction

Osteoporosis (OP) is a prevalent systemic metabolic bone disease characterized by low bone mass, microarchitectural deterioration of bone tissue, and consequent increase in bone fragility, thereby predisposing individuals to fractures (1). Furthermore, This condition has emerged as another major health threat to middle-aged and elderly populations following diabetes and hypertension, due to its high prevalence and potential risk of disabling complications, posing significant public health challenges comparable to chronic cardiovascular conditions (2). According to statistics, the number of individuals aged 60 and above diagnosed with osteoporosis in China has reached 264 million (approximately 18.7% of the total population), while the population aged 65 and above exceeds 190 million (accounting for about 13.5% of the total population). This makes China the country with the largest elderly population globally (3). The National Osteoporosis Epidemiological Survey reveals that the prevalence of OP among individuals aged 50 and older stands at 19.2%, with significant gender disparities: 32.1% in women compared to 6.9% in men. This rate escalates to 32.0% in populations aged 65 and above, demonstrating a pronounced sex-specific pattern where women exhibit 51.6% prevalence versus 10.7% in their male counterparts (4, 5). POP, the most prevalent form of OP, is classified into postmenopausal OP (Type I) and senile OP (Type II). Modern medicine has achieved comprehensive understanding of its pathogenesis and therapeutic strategies, with established clinical frameworks for disease management. Meanwhile, with ongoing research advancements in TCM-based POP prevention and treatment, traditional Chinese medicine (TCM) has accumulated substantial clinical experience in addressing this condition, offering safe and effective therapeutic alternatives for POP patients. This review article systematically examines the pathological mechanisms underlying POP and evaluates evidence-based TCM interventions, aiming to provide actionable insights for optimizing clinical decision-making in OP care.

## 2 Pathogenesis

Bone metabolic imbalance constitutes the central pathological process underlying OP. Bone metabolism refers to the dynamic equilibrium process involving continuous bone remodeling through osteoclastic bone resorption and osteoblastic bone formation, which is essential for maintaining skeletal health, structural integrity, and functional competence. This homeostatic regulation is maintained through the coordinated interaction between osteoblasts and osteoclasts. When the system demonstrates pathological deviations characterized by either hyperactivated osteoclast-mediated bone resorption or impaired osteoblast-driven bone formation, the bone metabolic homeostasis becomes dysregulated, thereby triggering the molecular cascade leading to OP pathogenesis.

### 2.1 Bone remodeling

Bone remodeling refers to the physiological process by which bone tissue morphology and density adapt in response to alterations in biomechanical environments. During skeletal maturation, growth and modeling activities essentially cease, while bone remodeling and turnover persist throughout life. Bone turnover at organ, tissue, and cellular levels manifests as physiological activities of bone cells, achieved through the remodeling process. Histologically, bone remodeling can be defined as the regenerative replacement of mature bone tissue through coordinated multicellular interactions, fundamentally representing a dual transformation mechanism encompassing both morphological and functional adaptations mediated by bone metabolic cell lineages. This process possesses dual regulatory significance: Firstly, it maintains biomechanical homeostasis of the skeletal system by cyclically eliminating progressive accumulations of microdamage in the bone matrix. Secondly, it achieves dynamic regulation of calcium-phosphorus metabolism and precise control of mineral deposition through the formation of structural bone units with appropriate mineralization levels. This multifunctional mechanism combining mechanical stress-adaptive remodeling and mineral metabolic equilibrium constitutes an essential safeguard for maintaining skeletal mechanical properties and mineral homeostasis.

Furthermore, studies have indicated that postmenopausal bone remodeling exhibits deeper resorption cavities, potentially attributable to prolonged osteoclast lifespan or reduced apoptosis (6). In senile OP, osteoblasts demonstrate diminished capacity to form new bone filling resorption cavities, manifested as age-related reductions in bone wall thickness and consequent decreases in bone volume. Postmenopausal OP is also characterized by reduced ratios of osteoblastic surfaces to osteoid tissue surfaces and decreased mineralization rates (7). Following OP onset, increased porosity subjects residual bone structures to amplified microdamage accumulation. This state initiates a vicious cycle: reduced bone mass increases mechanical fatigue damage in remaining bone tissue, subsequently activating remodeling processes that enhance bone resorption. These pathophysiological interactions further exacerbate bone loss and deteriorate bone quality, ultimately establishing a self-perpetuating “damage-resorption-redamage” cycle (8).

### 2.2 Hormone regulation

#### 2.2.1 Sex hormone

Sex hormones play a pivotal role in the pathogenesis of POP. During postmenopausal OP, decreased estrogen levels elevate bone remodeling activation rates, potentially inducing increased production of IL-6 and other cytokines linked to the proliferation of osteoclasts and osteoblasts (9). Current evidence indicates that estrogen not only promotes bone formation but also suppresses bone resorption. Clinical studies report that postmenopausal women or those undergoing ovariectomy exhibit significantly elevated levels of bone-resorptive cytokines, including interleukin-1 (IL-1) and tumor necrosis factor-alpha (TNF-α), secreted by blood monocytes compared to premenopausal women (10, 11). Interleukin-6 (IL-6), recognized as a potent osteoclastogenic stimulator, plays a critical role in bone loss (12). Additionally, some studies note elevated soluble IL-6 receptor (sIL-6R) levels in ovariectomized women. Emerging evidence suggests that IL-6 signaling, beyond traditional membrane receptor-derived pathways, crucially depends on signals mediated by the sIL-6R-glycoprotein 130 (gp130) complex (13). Although most soluble receptors act as antagonists, sIL-6R exhibits agonist properties and collaborates with IL-6 to activate gp130-expressing target cells. Studies utilizing specific antibodies to block IL-6 signaling demonstrate that such inhibition prevents trabecular bone loss in ovariectomized mice but fails to suppress cortical bone loss (14, 15). This underscores the essential role of IL-6 signaling in mediating trabecular bone loss under estrogen-deficient conditions.

The OPG/RANKL/RANK signaling pathway plays a critical role in regulating bone metabolism (16). RANK, a key cytokine inducing osteoclast differentiation and maturation, binds to RANKL on osteoclasts and their precursor cells, strongly stimulating osteoclast differentiation and maturation while inhibiting osteoclast apoptosis. Multiple studies have demonstrated that estrogen suppresses the RANKL/RANK signaling pathway (17). Research indicates that postmenopausal women in early stages exhibit higher RANKL expression in bone marrow mononuclear cells (MNCs) compared to premenopausal women (18). Nakamura et al. (19) revealed through knockout mouse models that estrogen activates estrogen receptor α in osteoblasts and osteocytes, upregulates OPG (a decoy receptor for RANKL), and inhibits RANKL production, thereby indirectly suppressing osteoclast activity. Studies show that 17-β estradiol stimulates RANKL and OPG protein expression in human osteoblasts within 24 hours; however, by 48 hours, RANKL protein levels return to baseline while OPG expression remains elevated, indicating that estrogen modulates the OPG/RANKL ratio to reduce osteoclastogenesis.

Studies on molecular regulatory mechanisms demonstrate that testosterone significantly reduces bone matrix degradation activity by negatively regulating the biosynthesis of receptor activator of nuclear factor kappa-B ligand (RANKL) and upregulating osteoprotegerin (OPG) production, thereby disrupting the signaling pathway between osteoclasts and osteoblasts (20). Furthermore, androgens and estrogen exhibit bidirectional metabolic interconversion mediated by specific aromatases. Androgens are aromatized to estrogen via CYP19A1 enzyme-catalyzed reactions, a process constituting a critical molecular pathway for sex hormone-regulated bone metabolism (21). This hormonal interconversion also reduces the risk of OP occurrence.

#### 2.2.2 Parathyroid hormone

Parathyroid hormone (PTH) exerts its physiological effects primarily by promoting bone resorption and turnover, mobilizing calcium from bone into the bloodstream to elevate serum calcium levels. PTH influences various types of bone cells through distinct mechanisms. Firstly, PTH induces an increase in osteoclast number and activity, accelerating bone resorption. Subsequently, osteoblast activity rises, driving accelerated bone metabolism and new bone formation. Secondly, PTH inhibits phosphate and bicarbonate (HCO3-) reabsorption in the renal proximal convoluted tubules, enhancing renal phosphate excretion and thereby reducing serum phosphate levels (22). furthermore, PTH stimulates calcium reabsorption in the distal convoluted tubules, lowering calcium concentration in the renal tubular lumen (23), while also promoting intestinal calcium absorption and reducing urinary calcium excretion.

**Figure 1:**
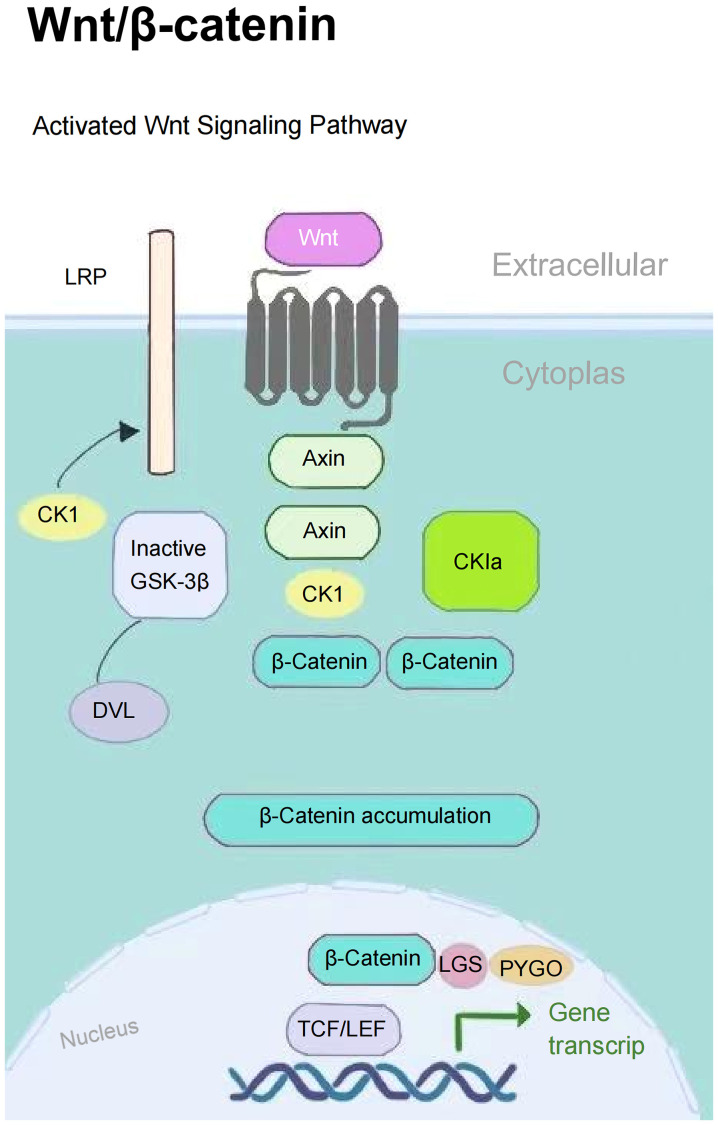
Activated Wnt signaling pathway.

The PI3K/AKT signaling pathway plays a pivotal role in biological metabolism, extensively regulating cellular metabolic activities and modulated by a series of growth factors and cytokines. This pathway enhances the osteogenic-angiogenic capacity of bone marrow mesenchymal stem cells (BMSCs), promoting osteoporotic bone repair (24). The Wnt/β-catenin signaling pathway inhibits β-catenin degradation through Wnt proteins, activates the expression of downstream osteogenesis-related genes, and directly regulates the osteogenic differentiation process (25) (**Figure 1**). These two pathways establish a complementary regulatory network through distinct molecular mechanisms, collectively constituting the core molecular foundation for maintaining skeletal metabolic homeostasis.

Intermittent PTH exerts anabolic effects by activating the PKA signaling pathway in osteoprogenitor cells, stabilizing β-catenin, promoting committed osteoblast precursor differentiation and osteocyte apoptosis to stimulate bone formation (26, 27). The therapeutic efficacy of low-dose intermittent PTH as a skeletal anabolic regimen has been well-documented in OP treatment (28). However, sustained elevation of serum PTH levels induces RANKL production in PTH-targeted cells, further promoting osteoclastogenesis and enhancing bone resorption (29).

While PTH is central to bone metabolism regulation, its structural and functional homolog, Parathyroid Hormone-related Protein (PTHrP), shares key receptors and plays critical, distinct roles in skeletal biology that warrant examination. PTHrP acts as a key hormonal regulator of bone formation, maintaining the dynamic equilibrium between bone resorption and formation through modulation of the RANKL/OPG balance (30). Karaplis et al. (31) observed that PTHrP knockout mice exhibited embryonic lethality or immediate postnatal death accompanied by severe skeletal dysplasia, unequivocally establishing PTHrP’s dominant role in bone metabolism. Additionally, Kovacs et al. (32) reported significantly reduced fetal blood calcium levels and impaired maternal-fetal calcium transport in PTHrP-deficient mice, indirectly resulting in defective skeletal mineralization. These findings collectively underscore the critical importance of PTHrP in osteogenesis.

#### 2.2.3 Active vitamin D

Vitamin D is an essential substance for skeletal development, maintenance, and the regulation of calcium-phosphate homeostasis. Vitamin D is initially hydroxylated in the liver to 25-hydroxyvitamin D_3_ [2-5(OH)D_3_], which serves as the predominant circulating form of vitamin D and the most reliable clinical biomarker for assessing vitamin D status. Loss-of-function mutations in the human vitamin D receptor (VDR) result in resistance to 1,25-dihydroxyvitamin D_3_ [1,25(OH)_2_D_3_], leading to hereditary vitamin D-resistant rickets type I. This condition is characterized by marked expansion of the hypertrophic chondrocyte zone, disorganized tissue architecture, and progressive enlargement of the epiphyseal growth plate (33). With adequate dietary calcium intake, 1,25(OH)2D3 ensures sufficient calcium supply by stimulating intestinal calcium absorption and renal calcium reabsorption, thereby indirectly modulating bone homeostasis and mineralization (34). Furthermore, 1,25(OH)2D3 targets multiple organs including the parathyroid glands and pancreas. Studies demonstrate that it suppresses PTH synthesis and secretion in the parathyroid glands (35), while enhancing insulin secretion from pancreatic β-cells (36), both of which exert regulatory effects on bone metabolism.

### 2.3 Genetic factors

The genetic predisposition to OP emerged in early research. Studies indicate that the heritability of bone mineral density (BMD) ranges from 50% to 85% (37), while the heritability of fracture risk is approximately 25%-35% (38). With the advent of genome-wide association studies (GWAS), scientists have identified hundreds of genetic loci associated with BMD. Notably, the vitamin D receptor (VDR) gene (39–41), low-density lipoprotein receptor-related protein 5/6 (LRP5/6) genes (42, 43), and parathyroid hormone (PTH) gene (44, 45) have been strongly linked to OP pathogenesis.

In addition to the aforementioned regulatory mechanisms, the pathophysiology of POP is also intricately linked to central nervous system (CNS) signaling pathways. These factors coordinately regulate osteoblasts and osteoclasts, thereby maintaining the dynamic equilibrium of bone tissue metabolism.

While these molecular mechanisms delineate the pathophysiology of POP through a biomedical lens, TCM offers a complementary etiopathogenic perspective rooted in systemic pattern differentiation. The following section explores how concepts such as ‘Kidney Deficiency,’ ‘Spleen Dysfunction,’ and ‘Meridian Stagnation’ in TCM correlate with and expand upon the preceding Western pathological models, providing a holistic foundation for integrative interventions.

## 3 Etiology and pathogenesis of TCM

Within the theoretical framework of TCM, there is no direct or explicit discussion regarding POP. Based on its core clinical manifestations and pathogenesis, POP can be categorized under “bone impediment (gubi)” and “bone wilting (guwei)”. As recorded in the “Chapter on Wilting Patterns” in the *Suwen* (*Basic Questions*): “When there is heat in the kidney qi, the lumbar spine fails to erect, resulting in desiccated bones and diminished marrow, thereby manifesting as bone wilting.” From a TCM perspective, POP is considered a complex disorder arising from dysfunction of multiple zang-fu organs and prolonged cumulative influences of various pathogenic factors. According to the *Expert Consensus on the Prevention and Treatment of Primary Osteoporosis with Traditional Chinese Medicine (2020)*, the pathogenesis of POP can be classified into four patterns: 1) deficiency of kidney essence, 2) weakness of the spleen and stomach, 3) liver depression and blood deficiency, and 4) blood stasis and qi stagnation (46) (**Table 1**).

**Table 1:** Traditional chinese medicine syndromes, pathogenesis, and associated modern mechanisms with representative herbs and formulas.

TCM syndromes	Core pathogenesis	Modern mechanisms	Representative herbs	Representative formulas
Deficiency of Kidney Essence	decline in the kidney’s function of storing essence and generating marrow	Decreased estrogen levels. Genetic aspects: VDR gene mutation → Disorder of calcium and phosphorus metabolism.	Epimedium Eucommia	Zuoguiwan Youguiwan
Weakness of the Spleen and Stomach	Spleen failing in transportation leads to insufficiency in the production and transformation of qi and blood	Nutritional malabsorption	Atractylodes Hawthorn	Buzhongyiqitang
Liver Depression and Blood Deficiency	Dysregulation of “the liver governs the sinews and stores blood” results in under-nourishment of the bone marrow.	Neuroendocrine disorders	Bupleurum Peony	Xiaoyaosan
Blood Stasis and Qi Stagnation	Impairment of qi and blood flow leads to localized stasis and obstruction, resulting in failure in nourishment of the bones	Microcirculatory disorders	Persicae Semen Corydalis Rhizome	Shentongzhuyutang

### 3.1 Deficiency of kidney essence

According to TCM theory, the waxing and waning of kidney qi (shenqi) is closely associated with the development and progression of POP. The discourse on “male eight and female seven cycles” in the *Suwen·Shanggu Tianzhen Lun Pian (Basic Questions·Chapter on Ancient Innocence)* exemplifies the concept that “the kidney serves as the foundation of innate constitution” (shenweixiantianzhiben). This clarifies the crucial role of kidney essence (shenjing) in regulating human growth, development, and reproductive functions. The kidney stores the essence (jing) of the entire body, governs the bones, and generates marrow. Under physiological conditions, the sufficiency of kidney essence serves as the fundamental source for the normal production and nourishment of bone marrow (47). With natural aging, the stored essence in the kidney gradually depletes, leading to insufficient resources for marrow generation and nourishment. Consequently, the bones and marrow lose effective sustenance, ultimately resulting in the pathological state of “desiccated bones and deficient marrow” (gukusuixu).

Wang et al. (48) extensively explored the pivotal roles of kidney essence and heavenly gui(tiangui) in bone marrow development. They proposed that deficiency of kidney essence and decline of tiangui constitute key pathological foundations for OP in postmenopausal women. This perspective underscores the indispensable role of kidney essence and tiangui in maintaining skeletal health. Li et al. (49) conducted a microscopic analysis through modern scientific approaches to reinterpret the TCM theory of “the kidney storing essence to generate marrow” (shencangjingshengsui). Their study provides novel scientific interpretations, suggesting that insufficiency of kidney essence not only represents a primary pathological mechanism of POP but also directly reflects the impairment of the kidney’s consolidating and storing functions (gusheyufengcang). These findings offer robust scientific validation for TCM theories at the microscopic level.

The concept of congenital essence (xiantianzhijing) in TCM, encompassing genetic information, life potential, and constitutional endowment, exhibits a remarkable congruence with modern genetic research on gene polymorphisms and their influence on bone metabolic programming. The constitutional basis of deficiency of kidney essence (shenjingkuixu) can be partially interpreted at the molecular level as carrying a susceptible gene combination unfavorable for bone mass accumulation and maintenance. The TCM concept of congenital insufficiency due to “parental weakness” (fumutiruo) or “insufficiency of the fetal origin” (taiyuanbuchong) corresponds to the Western medical understanding of inheriting genotypes associated with low peak bone mass (PBM) or high bone turnover, thereby increasing the risk of POP.

Furthermore, the state of “shen jing kui xu” leading to “bone failing to receive nourishment” (gushisuoyang) precisely mirrors the core pathological mechanism in Western bone metabolism where bone resorption exceeds bone formation. Deficiency of kidney essence corresponds to a diminished capacity of BMSCs for proliferation and differentiation into osteoblasts. Essence depletion results in weakened BMSCs vitality and a reduced number of osteoblast precursors, directly leading to a decreased bone formation rate and deterioration of bone matrix quality.

### 3.2 Weakness of the spleen and stomach

In TCM theory, the spleen (pi) is regarded as the “foundation of acquired constitution” (houtianzhiben) and the “source of qi and blood transformation and production” (qixueshenghuazhiyuan), playing a vital role in acquiring nutrients essential for sustaining life activities. Spleen failing in transportation (pishijianyun) is one of the pathological foundations of POP. As stated in the *Piwei Lun* (*Treatise on the Spleen and Stomach*): “When the spleen is diseased, its pathogenic influence descends to invade the kidney. Earth overcomes water, leading to bone weakness and fatigue, termed bone erosion (gushi).” Furthermore, the spleen is closely connected to the muscles and limbs, and its functional strength directly influences muscular development and limb mobility. The *Suwen·Taiyin Yangming Lun* (*Basic Questions·Chapter on Taiyin and Yangming*) notes: “If the spleen is diseased and fails to transport fluids for the stomach … the sinews, bones, and muscles will lack qi for nourishment, thereby losing their functional capacity. “With aging, spleen failing in transportation results in diminished production of qi and blood, insufficient essence, and impaired distribution of fluids (jinye) throughout the body. This leads to inadequate nourishment of the zang-fu organs, failure to replenish sinews, bones, and muscles, subsequent depletion of kidney essence (shenjingkuisun), insufficiency of essence and marrow (jingkuisuishao), bone desiccation due to marrow deficiency (suixuguku), and ultimately bone malnourishment, culminating in POP (50).

Song et al. (51) emphasize the intrinsic connection between the spleen-kidney system and the musculoskeletal structure. They highlight that spleen-kidney deficiency profoundly disrupts the coordination between bones and muscles. Thus, restoring the balance between muscles and bones through spleen-stomach regulation (piweitiaoli) holds significant therapeutic value in preventing and treating POP.

Weakness of the spleen and stomach leading to malabsorption of nutrient essence directly corresponds to the Western medical concepts of insufficient intake or malabsorption of key substances essential for bone construction and metabolism, such as calcium, phosphorus, protein, and vitamin D. Calcium is primarily absorbed via active transport in the duodenum and proximal jejunum, a process dependent on active vitamin D. Gastrointestinal disorders—including functional gastrointestinal disorders, insufficient gastric acid secretion, and intestinal mucosal damage—can significantly reduce calcium absorption efficiency. This consequently disrupts bone metabolic balance and ultimately contributes to the development of OP.

### 3.3 Liver depression and blood deficiency

Within the TCM theoretical system, the liver (gan) fulfills dual physiological functions: governing free coursing (zhunshuxie) and storing blood (cangxue). As stated in the *Suwen·Shanggu Tianzhen Lun* (*Basic Questions·Chapter on Ancient Innocence*): “At the age of seven or eight (for females and males, respectively), liver qi (ganqi) declines, leading to impaired mobility of the sinews.” When liver blood (ganxue) is abundant and its free coursing function operates normally, the sinews and vessels receive adequate nourishment, ensuring robust musculoskeletal health. Conversely, liver qi stagnation (ganqiyujie) disrupts qi and blood circulation, causing vessel congelation (jingmainingzhi) and restricted movement. The Zhu*bing Yuanhou Lun·Juan San·Xulao Bing Zhu Hou Xulao Shang Jingu Hou* (*Treatise on the Origins and Manifestations of Various Diseases·Volume 3·Consumptive Disorder Patterns: Consumptive Injury of Sinews and Bones*) states: “The liver governs the sinews and stores blood, while the kidney governs the bones and generates marrow. Chronic depletion of blood and marrow through consumptive disorders injures the sinews and bones”.

Wen et al. (52) further clarify that the liver and kidney share a common origin (ganshentongyuan), with essence and blood mutually generating each other to sustain marrow production. Sufficient marrow ensures strong sinews and bones; however, dysfunction in their storage and discharge capacities (cangxiegongneng) leads to essence insufficiency and marrow desiccation (jingkuisuiku), resulting in musculoskeletal degeneration. Zhang et al. (53) emphasize the critical role of emotional factors in POP pathogenesis. Liver failing in free coursing (ganshishuxie) induces liver depression (ganyu), obstructing qi movement and depleting yin-blood (yinxue), thereby depriving the marrow and vessels of nourishment. Thus, tonifying the liver and kidney (buyiganshen) is essential in treating OP.

In TCM theory, liver dysfunction in free coursing (ganshishuxie) is the key pathogenesis underlying emotional abnormalities. The smooth regulation of qi dynamic (qiji) forms the foundation for normal emotional activity. Stagnation of the qi dynamic inevitably obstructs the expression of emotions and constitutes the core pathology in the formation of depression syndrome. Research indicates that when individuals are in a state of emotional suppression, the hypothalamic-pituitary-adrenal (HPA) axis becomes hyperactivated, generating excessive glucocorticoids. These hormones directly inhibit osteoblast proliferation and differentiation. Furthermore, they promote osteoclast activity by upregulating RANKL expression and suppressing its decoy receptor, OPG, thereby disrupting the RANKL/OPG balance (54). Gebara et al. (55) also found, through research analysis, that depressive symptoms were significantly associated with reduced BMD.

Prolonged stagnation of the qi dynamic that fails to be resolved will eventually transform into heat and generate fire (yuerhuare), forming a “liver fire” (ganhuo) pattern. This manifests as emotional agitation, restlessness, and irritability. This process complements, at the pathophysiological level, the Western medical mechanism where sympathetic nervous system excitation triggers RANKL/OPG imbalance, ultimately leading to OP (56). This indicates that emotional well-being must also be addressed in the prevention and management of OP. TCM-specific therapies such as liver-soothing and fire-clearing therapies can be applied to achieve a holistic mind-body dual approach. This provides a solid theoretical foundation for constructing more comprehensive and individualized OP prevention and management strategies.

### 3.4 Blood stasis and Qi stagnation

In TCM theory, spleen (pi) and blood (xue) are regarded as two fundamental substances within the human body, holding paramount importance in sustaining life activities. Qi serves as the dynamic force for blood production and circulation, while blood acts as the material foundation and carrier for qi. This relationship is encapsulated in the axiom: “Qi is the commander of blood, and blood is the mother of qi” (qiweixuezhishuai, xueweiqizhimu). Both qi and blood are intimately linked to the sinews and bones. When their circulation is smooth and harmonious, the musculoskeletal system remains robust. As stated in *Yilin Gaicuo* (*Correcting the Errors in the Medical Forest*): “If primordial qi is deficient, it cannot reach the blood vessels. Vessels devoid of qi will stagnate and develop stasis.” Impeded circulation of qi and blood leads to qi stagnation and blood stasis (qizhixueyu), depriving the marrow of adequate nourishment and resulting in desiccated bones and diminished marrow (gukusuijian), which predisposes individuals to OP.

Jing et al. (57) propose that hemodynamic alterations, abnormal hemorheology, or changes in microvascular-related factors may induce microcirculatory disturbances (weixunhuanzhangai). Such disturbances obstruct the normal exchange of nutrients and oxygen among bone cells, impairing their growth and development. Prolonged microcirculatory dysfunction disrupts bone metabolism, ultimately contributing to the disease. A study on microcirculatory blood flow further indicates that these disturbances accelerate bone loss and hasten the onset of OP (58). Therefore, ensuring unobstructed qi and blood circulation is critical for preventing and managing POP.

The pathways for cellular material exchange depend on the blood supply provided by the capillary network, facilitated by the flow and diffusion of interstitial fluid within the canalicular network, and mediated by gap junctions between osteocytic processes. Microcirculatory disturbances directly lead to ischemia and hypoxia in bone tissue. Osteocytes are highly sensitive to hypoxia, which can induce osteocyte apoptosis. This provides a micro-pathological explanation for the TCM concept of “yu xue zu luo, gu shi suo yang” (static blood occluding the collaterals, resulting in bone failing to receive nourishment).

Within the TCM theoretical system, qi stagnation (qizhi) is a significant driver for the formation of static blood (yuxue). Conversely, the obstruction caused by static blood aggravates the impairment of qi dynamic (qiji), creating a vicious cycle termed blood stasis and qi stagnation. Simultaneously, static blood obstructing the luomai (collaterals/vessels) disrupts the distribution of jinye (fluids), leading to their extravasation. These extravasated fluids combine with extravasated blood and congeal, eventually transforming into phlegm-turbidity (tanzhuo). This ultimately results in a complex pathological state characterized by the intermingling of qi stagnation, blood stasis, and phlegm-turbidity.

In Western medical theory, microcirculatory disturbances activate the local innate immune system, triggering a sustained inflammatory cascade. Activated immune cells and damaged tissue cells release key pro-inflammatory cytokines in large quantities, including Tumor Necrosis Factor-alpha (TNF-α), Interleukin-1 (IL-1), Interleukin-6 (IL-6), and Interleukin-17 (IL-17), creating a “cytokine storm.” This disrupts bone metabolic balance.

In summary, TCM posits that the primary pathogenesis of OP lies in deficiency of the zang-fu organs (zangfuxusun), with kidney deficiency (shenxu) as the root cause, liver deficiency (ganxu) as a pivotal factor, spleen deficiency (pixu) as a significant contributor, and blood stasis (xueyu) as the pathological foundation. Scholarly studies indicate an intrinsic association between POP pathogenesis and TCM constitutional types (zhongyitizhi), underscoring the necessity to adhere to holistic principles (zhengtiguannian) and pattern differentiation-based treatment (bianzhenglunzhi) in clinical practice. This approach ensures the selection of tailored therapeutic strategies to address individual constitutional and pathological variations.

## 4 Treat

In clinical management, Western medical approaches to OP employ multidimensional interventions, with foundational treatment emphasizing the synergistic supplementation of calcium and vitamin D to maintain bone metabolic homeostasis. Anti-resorptive agents (e.g., bisphosphonates) mitigate bone loss by suppressing osteoclastic activity, while bone-forming agents (e.g., teriparatide) enhance osteoblastic function through targeted mechanisms (59, 60). However, certain pharmacological agents are associated with adverse effects during prolonged administration. For instance, hormone replacement therapy (HRT) and bisphosphonates, though effective in attenuating bone loss, carry risks such as atypical femoral fractures and elevated breast cancer incidence with long-term use (61, 62). This underscores the imperative to develop safer and more effective therapeutic strategies with minimized off-target effects.

The principle of TCM in treating POP is “pattern differentiation-based treatment, integration of disease and pattern, holistic regulation, and equal emphasis on prevention and treatment” (bianzhengshizhi, bingzhengjiehe, zhengtitiaojie, fangzhibingzhong). In clinical practice, formula prescription and herbal selection (qianfangyongyao) must be precisely tailored to the TCM patterns of POP. Clinicians should account for the chronicity of treatment, monitor shifts in patient patterns, and adjust prescriptions accordingly to achieve therapeutic goals such as alleviating clinical symptoms and delaying bone loss (46).

### 4.1 Traditional Chinese medicine monomers

#### 4.1.1 Epimedium

Epimedium (yinyanghuo), a herb widely utilized in TCM, possesses acrid, dispersing, sweet, tonifying, warm, and drying medicinal properties. It functions to tonify kidney yang (shenyang) to strengthen sinews and bones (qiangjingu), while expelling wind-dampness (qufengshi) and alleviating impediment pain (chubitong). As recorded in the Shennong Bencao Jing (Divine Farmer’s Classic of Materia Medica): “Epimedium primarily treats impotence (yinwei), severe injuries (jueshang), penile pain (jingzhongtong), promotes urination, boosts qi and physical strength (qili), and enhances mental vigor (qiangzhi)”.

Icariin (ICA), a primary bioactive constituent of *Epimedium*, demonstrates significant anti-osteoporotic efficacy. ICA has been validated to exert multifaceted pharmacological effects, including modulation of BMSCs, promotion of osteoblastic differentiation, and inhibition of osteoclastic function. Its mechanisms involve activation of signaling pathways such as Wnt/β-catenin and BMP-2/Runx2/OSX, as well as suppression of adipogenic differentiation-related factors like PPARγ. Studies confirm that ICA stimulates osteogenic differentiation of BMSCs to enhance bone formation while inhibiting osteoclastic differentiation and bone resorption activity (63).

In osteoporotic animal models, ICA downregulates pro-apoptotic proteins (e.g., Bax, caspase-3) and upregulates the anti-apoptotic protein Bcl-2, indicating its osteoprotective role via inhibition of osteocyte apoptosis (64, 65). Liang et al. (66) demonstrated through *in vitro* experiments and murine models that ICA enhances BMSC migration by upregulating CXCR4 expression and promotes osteogenic differentiation via ERK pathway activation.

In studies investigating fracture healing, ICA has been found to promote angiogenesis and ameliorate the bone regeneration microenvironment by enhancing vascular endothelial growth factor (VEGF) signaling (67–69). Recent studies further indicate that ICA mitigates OP through suppressing pro-inflammatory cytokine secretion (e.g., IL-6, TNF-α) in senescent macrophages, thereby resolving chronic inflammatory microenvironments (70, 71). Zhang et al. (72) demonstrated that ICA preferentially drives osteogenic differentiation of murine BMSCs while suppressing their adipogenic commitment via PPARγ downregulation, thereby enhancing bone formation and alleviating osteoporotic phenotypes.

#### 4.1.2 Eucommia ulmoides

Eucommia ulmoides (du zhong) demonstrates notable therapeutic efficacy in alleviating traditional symptom complexes, including lumbar and knee weakness and soreness, rheumatic arthralgia with stiffness, and aversion to cold with cold limbs (xinghanzhileng). Its pharmacological actions are particularly pronounced in addressing modern prevalent conditions such as lumbar degenerative diseases, OP, osteochondral pain, and chronic rheumatic disorders. Mechanistic studies suggest that these effects are mediated through dual modulation of bone metabolism equilibrium and anti-inflammatory pathways targeting TNF-α/NF-κB signaling cascades.

The primary active constituents in this medicinal material include pinoresinol diglucoside, geniposidic acid, aucubin, chlorogenic acid, and quercetin, among others. Studies indicate that constituents such as quercetin and aucubin promote alkaline phosphatase (ALP) activity in murine embryonic osteoblasts, stimulating osteoblast proliferation and differentiation (73). Quercetin maintains the viability of rat BMSCs, significantly upregulating ALP while downregulating the phosphorylation levels of PI3K, AKT, and mTOR. This suggests that quercetin may regulate BMSC viability and osteogenic differentiation via the PI3K/AKT/mTOR signaling pathway (74). Research also indicates that quercetin can inhibit osteoclast activation and reduce bone destruction by modulating the RANKL/RANK/OPG signaling pathway (75).

Eucommia ulmoides contains abundant polyphenolic compounds, such as flavonoids, which can inhibit BMD loss and improve trabecular bone parameters, thereby preventing the onset of POP (76). Furthermore, Eucommia ulmoides demonstrates beneficial effects in enhancing BMD, increasing bone hardness, and elevating serum levels of osteoblast markers, including alkaline phosphatase activity and osteocalcin (77), These findings collectively highlight its significant therapeutic potential for the prevention and treatment of POP.

#### 4.1.3 Drynariae rhizoma

Drynariae Rhizoma (Gusuibu), functions to tonify the kidney, strengthen bones, and promote wound healing to alleviate pain. Modern research demonstrates that *Drynaria* extract exhibits significant efficacy in treating POP, primarily mediated through the regulation of multiple metabolic pathways. Naringin and naringenin, the principal active constituents of Drynaria, show notable anti-osteoporotic effects.

Regarding the inhibition of osteoclast activity, Chen et al. (78) utilized a rat OP model established via retinoic acid gavage. Their findings showed that *Drynaria* extract increased BMD in the tibial metaphysis and accelerated the formation of early fibrous callus and cartilaginous callus. Concerning the promotion of osteoblast activity, Jin et al. (79) discovered that in retinoic acid-induced OP rats, naringin and naringenin significantly increased the number of trabecular bones and improved trabecular microarchitecture. Furthermore, in this process, naringin significantly upregulated the protein expression of ALP and parathyroid hormone 1 receptor (PTH1R). This suggests that *Drynaria* may promote osteoblast proliferation and prevent the onset of OP via the ALP and PTH1R pathways.

Regarding endocrine regulation, Drynariae Rhizoma can indirectly influence bone metabolism via modulation of estrogen. Guo et al. (80) demonstrated through *in vitro* cell proliferation assays that naringin and naringenin induce proliferation in estrogen-sensitive cell lines (MCF-7 cells) under estrogen-deficient conditions. This inductive effect exhibited concentration dependence, strengthening with increasing concentrations of Drynaria extract, indicating that naringin and naringenin possess estrogenic activity correlating with the concentration of Drynaria.

Dong et al. (81) co-cultured active constituents of Drynaria with rabbit BMSCs and subsequently implanted them into rabbits with calvarial defects. After 4 weeks, they observed significantly enhanced calvarial bone regeneration. They proposed that Drynaria controls osteoblast differentiation by inhibiting the binding of BMP-2 to bone morphogenetic protein receptors type-1A and -1B (BMPR-1A and BMPR-1B), thereby blocking BMPR-1A and BMPR-1B signal transduction. Lu et al. (82)found that total flavonoids from Drynaria may inhibit oxidative stress in ovariectomized rats and regulate skeletal homeostasis under physiological conditions, potentially through suppression of the Notch1/Hes1/Prdx1 signaling pathway.

#### 4.1.4 Prepared Rehmannia Root

Prepared Rehmannia Root (shudihuang) functions to tonify blood and enrich yin (buxueziyin), as well as boost essence to replenish marrow (yijingtiansui). As documented in the *Bencao Congxin* (*New Compilation of Materia Medica*), it “nourishes kidney water (zishenshui) and seals and fills the bone marrow (fengtiangusui)”.

Catalpol, one of the iridoid glycoside constituents of Rehmanniae Radix Praeparata, demonstrates significant anti-osteoporotic effects. Chen et al. (83) found that catalpol inhibits osteoclast differentiation by suppressing the expression of osteoclast-specific genes, thereby ameliorating OP. Their research also indicated that *Rehmanniae Radix Praeparata* not only improves OP by inhibiting osteoclast differentiation but also reduces bone loss by inducing osteoclast apoptosis. Furthermore, catalpol promotes the deacetylation of estrogen receptors, modulates fatty acid synthase ligand expression, and induces osteoclast apoptosis in rat OP models. These actions collectively reduce bone loss, increase BMD, and improve bone microstructure (84).

Regarding the regulation of molecular signaling pathways, Liu et al. (85) demonstrated that *Rehmanniae Radix Praeparata* can mitigate bone loss and improve bone quality in ovariectomized rats, at least partially through modulation of the canonical Wnt/β-catenin signaling pathway (85). Furthermore, *Rehmanniae Radix Praeparata* regulates bone metabolism via multiple pathways, including modulating the human endocrine system (86), altering ion channel activity (87), and influencing the metabolism of surrounding bone tissues (88).

Prepared Rehmannia Root demonstrates significant potential in anti-osteoporotic drug development. Its traditional functions of tonifying blood and enriching yin (buxueziyin) and boosting essence to replenish marrow (yijingtiansui) closely correlate with modern pharmacological anti-osteoporotic effects. Further research into the active constituents and mechanisms of action of Rehmanniae Radix Praeparata holds promise for providing more effective therapeutic options for patients with POP.

#### 4.1.5 Crataegus pinnatifida

While current research primarily focuses on the cardiovascular effects of *Crataegus pinnatifida* (Hawthorn), studies indicate its significant potential in maintaining bone metabolic homeostasis. As one of its active constituents, maslinic acid inhibits osteoclast differentiation, exerting bone-protective effects. Maslinic acid specifically blocks RANKL-induced phosphorylation of IκBα, thereby reducing the nuclear translocation efficiency of NF-κB and downregulating mRNA expression levels of the key osteoclast regulator NFATc1. It also modulates the activity of the MAPK signaling pathway. Collectively, these actions inhibit osteoclast differentiation and activation, reducing bone resorption (89).

Regarding osteoblast promotion, active components in hawthorn, particularly hawthorn flavonoids, significantly stimulate osteoblast proliferation. This occurs through the regulation of cell cycle-related proteins, such as upregulating Cyclin D1 and CDK4 expression, and activating the ERK signaling pathway (90). Furthermore, studies indicate that hawthorn enhances the mineralization capacity of osteoblasts. This is achieved by augmenting osteocalcin expression, increasing ALP activity, modulating intracellular calcium ion concentration and calcium signaling pathways, and promoting calcium salt deposition within the bone matrix (91).

The antioxidant and anti-inflammatory properties of hawthorn also contribute positively to OP management (92, 93). Constituents such as flavonoids, vitamin C, and maslinic acid exhibit potent antioxidant and anti-inflammatory activities. These components scavenge excessive free radicals, suppress the expression and release of inflammatory cytokines, inhibit osteoclast activity, and promote osteoblast function, thereby helping to maintain bone metabolic balance.

#### 4.1.6 Persicae Semen

*Persicae Semen* (Taoren) functions to lubricate the intestines for bowel movement and invigorate blood circulation to dispel stasis. As documented in *Ben Cao Gang Mu* (*Compendium of Materia Medica*), it “promotes blood circulation, moistens dryness, unblocks meridians, and alleviates pain.” Modern research indicates that *Persicae Semen* improves local microcirculatory disturbances by activating blood circulation and resolving stasis. When combined with kidney-tonifying herbs, it provides both symptomatic relief by ameliorating blood stasis and fundamental treatment by promoting bone tissue repair, embodying the therapeutic principle of “disperse stasis to regenerate new tissue”.

Amygdalin and flavonoids are its principal active constituents. Studies demonstrate that amygdalin inhibits osteoclast differentiation and promotes osteoblast formation by suppressing the NF-κB signaling pathway while activating the Wnt/β-catenin pathway. It also effectively alleviates inflammatory pain associated with OP (94). Flavonoids exhibit significant antioxidant activity (95), accelerating the differentiation of mesenchymal stem cells, osteoblasts, and osteocytes, thereby demonstrating beneficial effects against POP.

Current research indicates significant potential for TCM in treating POP. Beyond the aforementioned herbs, numerous other TCM agents demonstrate considerable promise in managing OP clinically, such as Salvia miltiorrhiza (Danshen) and Ligusticum (chuanxiong). These herbs exert positive therapeutic effects on OP through distinct mechanisms of action (**Table 2**). For instance, *Danshen*, containing active components like tanshinones, promotes blood circulation and improves the bone microenvironment (96). Chuanxiong, rich in active compounds such as ligustrazine and ferulic acid, regulates bone metabolism and reduces bone loss (97). With ongoing research, TCM is expected to expand its role significantly in the therapeutic landscape of POP in the future.

**Table 2:** Pharmacological mechanisms and key active constituents of representative herbs for bone-related conditions.

Representative herbs	Main active constituents	Mechanisms
Epimedium	Icariin (ICA)	① Activates Wnt/β-catenin and BMP-2/Runx2/OSX pathways to promote osteogenic differentiation of BMSCs ② Suppresses PPARγ to inhibit adipogenic differentiation ③ Downregulates Bax/caspase-3 and upregulates Bcl-2 to inhibit osteocyte apoptosis ④ Enhances BMSC migration via CXCR4 upregulation
Eucommia ulmoides	Quercetin, Aucubin	① Activates PI3K/AKT/mTOR pathway to promote BMSC osteogenesis ② Modulates RANKL/RANK/OPG pathway to suppress osteoclast activation ③ Elevates osteocalcin levels and BMD ④ Flavonoids improve trabecular microstructure
Drynariae Rhizoma	Naringin, Naringenin	① Concentration-dependent estrogen-like activity (induces MCF-7 cell proliferation) ② Upregulates ALP and PTH1R expression to stimulate osteoblast proliferation ③ Blocks BMP-2 binding to BMPR-1A/1B to regulate osteogenesis ④ Suppresses oxidative stress via Notch1/Hes1/Prdx1 pathway
Prepared Rehmannia Root	Catalpol	① Downregulates osteoclast-specific genes to induce apoptosis ② Promotes osteoclast apoptosis via Sirt6-ERα-FasL axis ③ Activates Wnt/β-catenin pathway to reduce bone loss ④ Modulates endocrine system and calcium channels
Crataegus pinnatifida	Maslinic acid, Flavonoids	① Blocks RANKL-induced IκBα phosphorylation, inhibits NF-κB nuclear translocation and NFATc1 expression ② Upregulates Cyclin D1/CDK4 and activates ERK pathway to promote osteoblast proliferation ③ Regulates calcium signaling to enhance bone matrix mineralization ④ Scavenges free radicals and suppresses inflammatory cytokines
Persicae Semen	Amygdalin, Flavonoids	① Inhibits NF-κB pathway to alleviate bone inflammatory pain ② Activates Wnt/β-catenin pathway to promote osteogenesis ③ Flavonoids accelerate osteogenic differentiation of mesenchymal stem cells
Salvia miltiorrhiza	Tanshinone, Salvianolic acid	① Promotes blood circulation and improves bone microenvironment ② Inhibits RANKL-induced osteoclast formation via suppressing MAPK/NF-κB signaling pathways ③ Reduces inflammatory bone destruction
Ligusticum	Ligustrazine, Ferulic acid	① Regulates bone metabolism balance ② Inhibits osteoclast-mediated bone resorption by downregulating RANKL expression ③ Enhances bone microcirculation and angiogenesis

### 4.2 Traditional Chinese medicine compounds

TCM compound formulas exemplify the core therapeutic principles of syndrome differentiation and treatment (bianzhenglunzhi) and holistic correlation (zhengtiguannian), as well as the holistic concept. In the clinical treatment of POP, several classic formulas are extensively utilized, including Qiangguyin, Liuweidihuangwan, Zuoguiwan, Youguiwan, etc.

#### 4.2.1 Qiangguyin

Qiangguyin (QGY) is an empirical formula for treating POP. It employs the therapeutic principle of “augmenting qi and warming the channels” (yiqiwenjing), which centers on tonifying the liver, spleen, and kidney (buyiganpishen) while incorporating herbs that warm the channels and free the collaterals (wenjingtongluo), activate blood and alleviate pain (huoxuezhitong), thus achieving the purpose of treating OP.

Zhang et al. (98) demonstrated that QGY may exert anti-osteoporotic effects by inhibiting complement C3 expression. Other researchers have indicated that its mechanism of action against POP involves influencing the expression and release of inflammatory factors in OP model rats via the RANKL/RANK/NF-κB/NLRP3 inflammasome pathway, thereby further affecting osteoclast differentiation and regulating bone metabolic balance (99). Concurrently, QGY can also interfere with the binding of CKIP-1 to AKT, a key factor in the PI3K/AKT/mTORC2 signaling axis. By impacting AKT activation and disrupting upstream signaling to mTOR, QGY ultimately activates the PI3K/AKT/mTORC2 signaling pathway, leading to downregulation of autophagy levels and subsequent maintenance of bone homeostasis (100). Furthermore, Wen et al. (101) discovered that QGY suppresses the overexpression of Fatty Acid Binding Protein 4 (FABP4) and Peroxisome Proliferator-Activated Receptor Gamma (PPARγ), while also inhibiting p38 expression to prevent lipid accumulation, thereby achieving anti-osteoporotic effects. Clinically, QGY demonstrates significant efficacy with no apparent adverse effects reported (102, 103). These findings collectively indicate that QGY holds substantial promise as a therapeutic agent for POP.

#### 4.2.2 Liuweidihuangwan

Liuweidihuangwan (LWDHW), a classic formula in TCM, functions to enrich yin and tonify the kidney (ziyinbushen). It is clinically applied for patterns of kidney yin deficiency (shenyinxu) manifesting as lumbar and knee soreness with weakness (yaoxisuanruan) and related symptoms.

Zhao et al. (104) demonstrated that LWDHW promotes the expression of osteogenic differentiation proteins, such as Collagen I and RUNX2, and enhances ALP activity in MC3T3-E1 cells by activating the Wnt/β-catenin signaling pathway, offering a novel approach for POP treatment. Other researchers have indicated that LWDHW exerts anti-osteoporotic effects by inhibiting KDM7A and modulating the Wnt/β-catenin pathway (105, 106). Clinically, Tan et al. (107) found that LWDHW combined with zoledronic acid significantly improved clinical outcomes in POP patients, reducing pain intensity, enhancing lower back mobility and quality of life, and improving BMD. Further supporting its efficacy, Li et al. (108) confirmed the significant benefits of LWDHW in POP management, reporting that the treatment group receiving LWDHW combined with conventional Western medicine demonstrated superior improvements in BMD and bone turnover markers compared to conventional treatment alone. These collective findings indicate that LWDHW possesses the ability to inhibit bone resorption, increase BMD, and thus promote bone formation.

#### 4.2.3 Zuoguiwan

Zuoguiwan (ZGW), a seminal formula in TCM, primarily functions to enrich yin and tonify the kidney (ziyinbuxue). It demonstrates significant therapeutic efficacy for symptoms including lumbar and knee soreness with weakness (yaoxisuanruan) arising specifically from kidney yin insufficiency (shenyinbuzu).

Modern pharmacological research indicates that ZGW contains several bioactive chemical constituents crucial for improving POP, including quercetin, kaempferol, and phenylalanine (109). Liu et al. (110), utilizing an ovariectomized (OVX) rat model of OP, suggested that ZGW may exert its effects by reducing the mRNA and protein expression of the β2-adrenergic receptor (β2AR), downregulating RANKL signaling to reduce osteoclast differentiation, and concurrently upregulating osteoprotegerin (OPG) to enhance bone protection. Further studies demonstrate that ZGW can modulate the Orexin-A/Orexin receptor system to improve trabecular bone microstructure in OVX-induced osteoporotic rats, contributing to its anti-osteoporotic activity (111). Clinically, Kang et al. (112) conducted a study involving 150 POP patients with kidney yin deficiency. Patients were randomized into a control group receiving Caltrate D tablets (calcium carbonate/vitamin D) and a ZGW group receiving Caltrate D plus ZGW. After six months of treatment, the ZGW group achieved significantly greater relief from osteoporosis-related pain compared to the control group. This combined therapy not only effectively ameliorated the patients’ osteoporotic condition but also significantly increased BMD. These findings strongly demonstrate the potential benefits of ZGW for treating POP patients with kidney yin deficiency and provide robust clinical evidence supporting the application of TCM compound formulas in POP management.

#### 4.2.4 Youguiwan

Youguiwan (YGW), a foundational formula in TCM, functions to warm and tonify kidney yang (wenbushenyang). It is clinically indicated for patterns of kidney yang insufficiency (shenyangbuzu) manifesting as lumbar and knee soreness with coldness (yaoxisuanleng) and related symptoms.

Pharmacokinetic analysis indicates that active constituents from YGW rapidly enter systemic circulation and reach the target site to exert therapeutic effects. Furthermore, YGW exhibits a relatively stable metabolic profile with a prolonged elimination half-life, suggesting sustained drug action and durable therapeutic benefits for patients (113, 114). Studies demonstrate that YGW may promote osteogenic differentiation and improve bone tissue microstructure in OVX rats by activating the BMP-2/Smad signaling pathway, ultimately increasing BMD (115). Clinically, Liang et al. (116) conducted a randomized controlled trial involving 81 postmenopausal women with osteopenia and kidney yang deficiency. Participants were allocated to either a control group (receiving conventional treatment) or a treatment group (receiving conventional treatment plus YGW). After 12 weeks of intervention, the YGW add-on therapy demonstrated significant efficacy in managing kidney yang deficiency symptoms in postmenopausal osteopenia patients. It effectively reduced TCM syndrome scores, increased BMD, regulated bone metabolism, and improved quality of life, with a favorable safety profile and fewer adverse events. These results indicate that the combined regimen offers superior therapeutic outcomes compared to conventional Western treatment alone.

#### 4.2.5 Bushenhuoxue Decoction

Bushenhuoxue Decoction (BSHX) functions to tonify the kidney and strengthen sinews (bushenzhuangjin) while activating blood to alleviate pain (huoxuezhitong). It is clinically employed for OP resulting from kidney deficiency with blood stasis (shenxuxueyu).

Research indicates that BSHX promotes the osteogenic differentiation of BMSCs by increasing the expression of Hedgehog signaling-related genes Ihh, Gli2, and Runx2, thereby contributing to OP treatment (117). Luo et al. (118) further demonstrated that BSHX upregulates the expression of angiogenic factors in femoral head tissues of model rats, enhancing the angiogenic capacity of rat BMSCs, which partially underlies its anti-osteoporotic effects. Clinically, Wang et al. (119) conducted a controlled study involving 120 patients with knee osteoarthritis (KOA) comorbid with OP. Patients were randomized into two groups: a control group receiving conventional treatment and an observation group receiving conventional treatment plus BSHX. Following a three-month treatment period, BSHX was confirmed to significantly improve clinical symptoms and modulate bone metabolism markers in patients with KOA and comorbid OP, underscoring its unique therapeutic value and application potential in POP management.

### 4.3 Combination of traditional Chinese and Western medicine for prevention and treatment

TCM emphasizes a holistic approach. Through the comprehensive synthesis of the four diagnostic methods—inspection, auscultation, olfaction, inquiry, and palpation (si zhen he can), it focuses on individualized treatment. Non-invasive therapies such as Chinese herbal medicine, acupuncture, and therapeutic massage are employed to regulate yin and yang (tiaohe yinyang), dredge the channels and collaterals(shutong jingluo), and tonify qi and blood (tiaoli qixue), thereby alleviating OP symptoms.

Western medicine, conversely, prioritizes in-depth investigation into etiology and pathological mechanisms. It utilizes diagnostic tools including imaging, BMD, and biochemical markers to establish a clear diagnosis. Treatment modalities encompass pharmacological therapy, physical therapy, and surgical intervention, aiming to restore bone metabolic balance, increase BMD, and prevent fractures.

Although differences exist between TCM and Western medicine in their understanding and management of OP, the two approaches are not mutually exclusive but rather complementary and mutually reinforcing. TCM’s holistic perspective and individualized treatment principles can offer novel insights and therapeutic strategies for Western medicine. Conversely, modern scientific technologies and research methodologies of Western medicine can provide robust scientific validation and platforms for TCM research. Therefore, in the prevention and management of OP, it is essential to fully leverage the respective strengths of both systems. Strengthening the integration of Chinese and Western medicine to establish a new model featuring complementary advantages and synergistic effects will provide patients with more comprehensive and effective treatment strategies.

## 5 Prospects and shortcomings

As research into the pathogenesis of POP deepens, the unique advantages of TCM in its management are increasingly recognized, with studies indicating its broad therapeutic potential. However, contemporary TCM research in OP still faces significant challenges that require more in-depth exploration.

One of the major challenges is the difficulty in objectifying and standardizing TCM syndromes. TCM diagnoses are often based on subjective symptoms and signs, which can vary between practitioners and regions. This lack of uniform standards makes it difficult to compare and integrate research findings. Future research could focus on developing objective biomarkers for TCM syndromes using modern technologies such as imaging, laboratory tests, and bioinformatics. For example, potential biomarkers could be identified through multi-omics analyses of blood or tissue samples from patients with different TCM syndromes, providing a more objective basis for syndrome differentiation.

Another significant challenge lies in the complexity of TCM compound formulas. TCM treatments typically involve the use of compound formulas containing multiple herbs, each with several bioactive components. The interactions between these components can lead to synergistic or antagonistic effects, making it difficult to determine the specific mechanisms of action. To address this, more advanced techniques such as systems biology and network pharmacology could be employed. These approaches can help map the complex interactions between different components in TCM formulas and their targets in the body, providing a more comprehensive understanding of their therapeutic effects.

There is also a notable lack of high-quality clinical research in TCM for OP. Many studies suffer from small sample sizes, single-center designs, and a lack of rigorous controls, which limits the reliability and generalizability of their findings. More large-scale, multi-center, randomized controlled trials (RCTs) are needed to evaluate the efficacy and safety of TCM interventions. These studies should adhere to strict methodological standards, including proper randomization, blinding, and control group selection, to ensure the validity of the results. Additionally, research could explore new trial designs, such as adaptive clinical trials or real-world studies, to better reflect the clinical practice of TCM and improve the applicability of the findings.

Furthermore, the integration of TCM and Western medicine in the treatment of POP is still in its early stages, with an immature theoretical framework. While both TCM and Western medicine have their own advantages in managing POP, combining them requires a deeper understanding of their respective mechanisms and how they can complement each other. Future research could focus on developing a more comprehensive theoretical framework for integrating TCM and Western medicine in POP treatment. This could involve exploring the synergistic effects of TCM and Western medical interventions at the molecular and cellular levels, as well as developing new treatment strategies that combine the strengths of both approaches.

In terms of specific research directions, utilizing multi-omics technologies to investigate the molecular basis of specific TCM syndromes is a promising avenue. By analyzing the genetic, proteomic, and metabolomic profiles of patients with different TCM syndromes, researchers can identify potential molecular markers and pathways associated with these syndromes. This could provide a more scientific basis for TCM syndrome differentiation and personalized treatment.

Conducting RCTs based on the combination of disease and syndrome is another important direction. These trials could evaluate the efficacy of TCM interventions in patients with specific TCM syndromes, providing higher-quality evidence for TCM treatments. Additionally, efforts could be made to construct predictive models or efficacy evaluation systems that combine TCM and Western medicine for POP. Such models could integrate clinical data from both TCM and Western medical perspectives to predict disease progression and treatment outcomes, helping to optimize treatment strategies.

Finally, a deeper exploration of the multi-target mechanisms of classical TCM formulas is needed. By combining experimental studies and clinical research, the specific targets and pathways through which these formulas exert their effects can be identified. This could lead to the development of more effective TCM interventions for POP and provide new insights for drug development.

In summary, while TCM shows great potential in the management of POP, addressing the current research challenges requires more in-depth, specific, and methodologically sound studies. By focusing on the objectification of TCM syndromes, the mechanisms of compound formulas, high-quality clinical research, and the integration of TCM and Western medicine, we can advance the field and provide better treatment options for POP patients.
